# Establishment and utility assessment of posterior reversible encephalopathy syndrome early warning scoring (PEWS) scale establishment and utility assessment of PEWS scale

**DOI:** 10.1186/s12883-019-1247-0

**Published:** 2019-02-21

**Authors:** Li-Ping Zou, Li-Ying Liu, Hui Li, Yang-Yang Wang, Ying Liu, Jing Chen, Lin-Yan Hu, Meng-Jia Liu, Meng-Na Zhang, Qian Lu, Shu-Fang Ma

**Affiliations:** 1 Department of Pediatrics, Chinese PLA General Hospital, Beijing, 100853 People’s Republic of China; 2 Center of Epilepsy, Beijing Institute for Brain Disorders, Beijing, 100069 China; 3 Key Laboratory of Pediatric Hematology & Oncology, Ministry of Health, Department of Hematology and Oncology, Shanghai Children’s Medical Center, Shanghai Jiao Tong University School of Medicine, Shanghai, 200127 China

**Keywords:** Posterior reversible encephalopathy syndrome, Electroencephalogram, Seizure, Prevention, Scale

## Abstract

**Background:**

Posterior reversible encephalopathy syndrome (PRES) is a complication that occurs during various diseases’ treatment. Imaging examination is the gold standard for diagnosis. PRES frequently occurrence in patients with hematological malignancies results in poorer prognosis and higher mortality. We aim to establish a practical and operable scale for early prediction, assessment of the severity of the Posterior Reversible Encephalopathy Syndrome, and timely intervention for better prognosis.

**Methods:**

The scale designed by reviewing the literature and by referring to clinical practice. We assessed the reliability and validity of the scale. Scale-based assessment of children undergoing chemotherapy for acute lymphoblastic leukemia conducted as early warning and intervention for those who may have PRES.

**Results:**

Establishment of Posterior Reversible Encephalopathy Syndrome early warning scoring (PEWS) scale included three parts, as follows: (1) risk factors, including underlying disease, hypertension, Infection, and drug toxicity; (2) clinical features, including high cranial pressure, visual symptoms, seizure, and disturbance of consciousness; and (3) EEG features, including slow wave and epileptiform discharges. Utility assessment of PEWS scale showed that in 57 patients with acute lymphoblastic leukemia, 54 scored less than 10 and none of them detected as PRES. The other two had scores of 12 and 13 both diagnosed with PRES by brain MRI scan.

**Conclusions:**

PEWS scale can predict PRES early. PRES was highly suspected when the score was 10 points and more. Thus, prophylactic intervention can give to improve the prognosis of PRES.

## Background

Posterior reversible encephalopathy syndrome (PRES) has drawn the attention of clinicians in recent years. PRES was first discovered by Hinchey in 1996 as a clinical imaging syndrome [1]. The manifestations of PRES include epilepsy, headache, motor or mental disorders, and visual impairment. PRES mostly observed in patients with hypertension, eclampsia, renal dysfunction, bone marrow transplantation followed by immunosuppressant therapy, and hematologic malignancies after chemotherapy [2]. Most studies on PRES published before 2010 are case reports. We previously found that posterior reversible encephalopathy syndrome in patients with hematologic tumor confers worse prognosis. All the PRES patients with hematologic tumor had worse prognosis than those without hematologic tumor [3].

Large-sample retrospective studies about PRES in the past 2 years mainly focused on disease spectrum, pathogenesis, atypical lesion, and complications [4]. These studies suggest two possible kinds of pathophysiological of PRES. One mechanism is when a burst of arterial blood pressure exceeds the limit of self-regulation of the cerebrovascular system, the blood vessels dilate, and the blood–brain barrier destroyed via elevated cerebral perfusion pressure [5]. In the other mechanism, the blood–brain barrier will also injured when endothelial cells of the cerebrovascular space injured by immune suppressants, chemotherapy, and inflammatory causes [4, 6, 7]. Blood–brain barrier impairment leads to vasogenic brain edema because of leakage of plasma and blood cells from the cerebrovascular space to the intercellular space. The sympathetic nervous system is important for self-regulation of the cerebrovascular system. Vasogenic brain edema in PRES restricted to the posterior part of the brain probably because of the lack of regulation of the sympathetic nervous system in that region [4]. Reversible clinical and imaging manifestations can also explained by vasogenic brain edema. However, the association between lesion distribution and pathogenesis still unknown. We hypothesize that lesions associated with self-regulation of the cerebrovascular system usually restricted to the posterior brain, and the distribution of lesions resulting from chemical endothelial injury often also includes anterior brain regions.

Imaging examination is the gold standard for PRES diagnosis. Characteristic MRI results include symmetrical hypointense T1 signal, hyperintense T2 signal, and isointense or slightly hyperintense DWI signal in the posterior parietal lobe and occipital lobe. Studies in the past 2 years indicated that PRES can occur in areas beyond the posterior brain region, such as frontal lobe, temporal lobe, cerebellum, basal ganglia, brainstem, and the entire brain. PRES is not always reversible. Many sequelae, like cerebral hemorrhage, cerebral infarction, focal gliosis, brain atrophy, and cerebral necrosis, were observed when vasogenic brain edema develops into cytotoxic brain edema [8].

Cohort studies showed the presence of PRES in patients with hematological malignancies resulted in poorer prognosis and higher mortality, especially after hematopoietic stem cell transplantation (HSCT) [3, 9]. We need a practical and operable early warning scale for the early diagnosis and treatment of PRES.

## Methods

### Part one: Retrospective analysis and establishing PEWS scale

#### Patients

Thirty-one children diagnosed with PRES from 1 January 2001 to 1 April 2013 in Chinese PLA General Hospital, PLA Army General Hospital, Peking Union Medical College Hospital, Xin Hua Hospital affiliated to Shanghai Jiao Tong University School of Medicine, and Yu Ying Children’s Hospital of Wenzhou Medical University enrolled in this study. This research performed by the Declaration of Helsinki, and told consent existed from each patient’s parents or caregivers before the trial-related procedures. The criteria include 1) a manifestation of acute neurological syndromes including headache, visual changes, seizures, and altered mental status; and 2) cerebral magnetic resonance imaging (MRI) showed angioedema in the posterior region of the brain.

#### Data collection

The clinical information of patients established by searching for “posterior reversible encephalopathy syndrome” and “PRES” in five cooperative medical centers. Clinical features of all the objects, including initial symptoms, risk factors (e.g., hypertension, infection, and application of chemotherapeutic agents or immunosuppressive agents), drugs, and prognosis assessed. Symptoms recorded including headache, seizure, and visual changes. MRI reconfirmed by radiologists to analyze lesion distribution and involvement.

### Part two: Prospective study and the use of PEWS scale

#### Study design

Electroencephalogram (EEG) imaging involved awake and complete sleep (2 h to 4 h) states. EEG performed in children diagnosed with acute lymphoblastic leukemia by the MICM criteria for the initial time-induced chemotherapy (CCLG-ALL2008 scheme), before chemotherapy (D1), 15th day of chemotherapy (D15), and at the end of chemotherapy. All the patients routinely underwent sleep deprivation the night before the EEG examination. One or two parents of each patient accompanied their children during hospital stay as observers. Clinical manifestations and laboratory test results recorded in a sheet (disease diary) with the help of health care providers. These data also logged into a medical record. Symptoms recorded headache, seizure, and visual changes. After each EEG, the patients would reassessed by a scale, and those with a score of 10 or more would undergo brain MRI for PRES diagnosis. Early preventive chemotherapy adjustment program included the following: 1) suspension of Methotrexate intrathecal injection and vincristine administration for one time; and 2) dexamethasone decrement earlier than scheduled. *Statistical analysis*: Data analyzed using SPSS 17.0. Chi-square test or Fisher’s exact test used to assess the classified variables. Mann-Whitney U test or Wilcoxon test used to assess continuous variables. Age described as median age (Q1–Q3).

## Results

### Part one: Retrospective analysis and establishing PEWS scale

#### Patients’ characteristics

We recruited 31 patients diagnosed with PRES, which consisted of 16 males and 15 females. The median age of the patients was 7 years old (range 3–12 years), and 74.2% of the patients were younger than 10 years old. Only 4 of 29 patients experienced seizure once. Other patients experienced multiple episodes, and the maximum number of seizure attacks was > 10 times. After the onset of PRES, 9 patients presented notable confusion, and 4 patients were in a coma. The initial symptom for 23 patients (74.2%) was seizure. For 6 patients (19.4%), the first symptom was confusion. For 2 patients (6.5%), the initial symptom was visual impairment. The clinical symptoms and prognosis of patients with and without hematologic malignancies listed in Table 1.

**Table 1:** Clinical characteristics and prognosis of hematologic malignancies and non - hematologic malignancies

			Non- hematologic malignancies (*n* = 17)	Hematologic malignancies (*n* = 14)	*p*-value
sex (male/female)			8/9	8/6	
symptoms	seizure		16 (94.1%)	13 (92.9%)	1.000
	headache		11 (64.7%)	8 (57.1%)	0.724
	visual impairments	blurred vision	8 (47.1%)	7 (50%)	1.000
		blind	2 (11.8%)	0	0.488
		cortical blindness	1 (5.9%)	2 (14.3%)	0.576
	confusion		9 (53.0%)	4 (28.6%)	0.275
	High blood pressure		12 (70.6%)	7 (50%)	0.288
Abnormal brain imaging	Occipital lobe		13 (76.5%)	11 (78.6%)	1.000
	Parietal lobe		13 (76.5%)	10 (71.4%)	1.000
	Frontal lobe		7 (41.2%)	6 (42.9%)	1.000
prognosis	Complete remission		16 (94.1%)	9 (64.3%)	0.101
	Partial remission		0	3 (21.4%)	
	death		1 (5.9%)	2 (14.3%)	

By comparing the clinical characteristics and prognosis of children with PRES in hematologic malignancies and other diseases, we found no statistical difference in the results of the above-mentioned two groups of patients. However, partial remission and death patients in hematologic malignancies group were higher than that in the non-hematological malignancies group. The small sample size resulted in the lack of statistical difference. We concluded that children with hematological malignancies might present poor prognosis.

#### EEG examinations

Seventeen patients with PRES showed slow-wave activities in the background. Both δ and θ activities were found in 9 (52.9%) patients. In three cases, slow wave detected in the occipital region. The same finding found in four cases with epileptic discharges. The EEG of three patients (17.6%) suggested θ activity in the occipital region. Diffused δ activity observed in five patients (29.4%), among which one patient presented epileptic discharges.

Among 17 patients, 6 (35.3%) presented abnormal EEG in the occipital area. Eleven patients showed a diffused slow wave in the EEG background, among which five (45.5%) cases confined to the occipital and parietal lobes by MRI, and others (54.5%) affected in the frontal and temporal lobes (Table 2).

**Table 2:** characteristics of EEG in 17 children with PRES

results	number	percent
θ activity in occipital	3	17.6%
δ and θ activity in occipital	3	17.6%
Diffuse δ and θ activity	2	11.8%
Diffuse δ activity	4	23.5%
epileptic discharge + diffuse δ and θ activity	4	23.5%
epileptic discharge + diffuse δ activity	1	5.88%

#### Establishment of PEWS scale

The PEWS scale consists of the three following aspects: high-risk factors, clinical characteristics, and EEG features. All criteria derived from the literature or clinical experience. To reduce the influence of diagnostic criteria for the inappropriate performance of a patient in the literature on the whole scale, we restricted the scores for each part of the scale.

The maximum scores for the risk factors, clinical features, and EEG features were 10, 10, and 6, respectively, for a maximum total score of 26 points. (Table 3).

**Table 3:** PEWS scale

risk factor (maximum:10 points)			clinical features (maximum:10 points)			EEG features (maximum:6 points)		
Underlying disease (maximum: 3 points)	Hematologic malignancies	3	High cranial pressure (maximum: 1 points)	Headache	1	Slow wave (maximum: 3 points)	Focal θ activity	1
	Autoimmune disease	2		Nausea, vomiting	1		Focal δ activity	2
	Other diseases	1	Visual symptoms (maximum: 3 points)	Blindness or blurred vision	2		Focal δ and θ activity	2
Hypertension (maximum: 3 points)	Level1(< 159/ 99)	1		Cortical blindness	3		Diffused θ activity	2
	Level2 (< 179/109)	2	Seizure (maximum: 3 points)	Generalized seizures	3		Diffused δ and θ activity	3
	Level3 (> 180/110)	3		Partial seizures	2		Diffused δ activity	3
Infection (maximum: 2 points)	Mild infection	1		Automatism	1	Epileptiform discharges (maximum: 3 points)	Focal discharge	2
	Septicemia	2	Disturbance of consciousness (maximum: 3 points)	Confusion	2			
Drug toxicity (maximum: 2 points)	Cytotoxic drugs	2		Coma	3		widespread discharge	3

In our study, two groups of patients evaluated using the PEWS scale. Seventeen PRES cases with a median age of 12 years old (8 males and 9 females) and 15 NON-PRES cases with a median age of 9.5 years old (9 males and 6 females; 11 patients with viral encephalitis and 4 patients with lupus encephalopathy), all with age-matched hospitalization, enrolled. All the patients completed radiology and EEG.

**Fig. 1:**
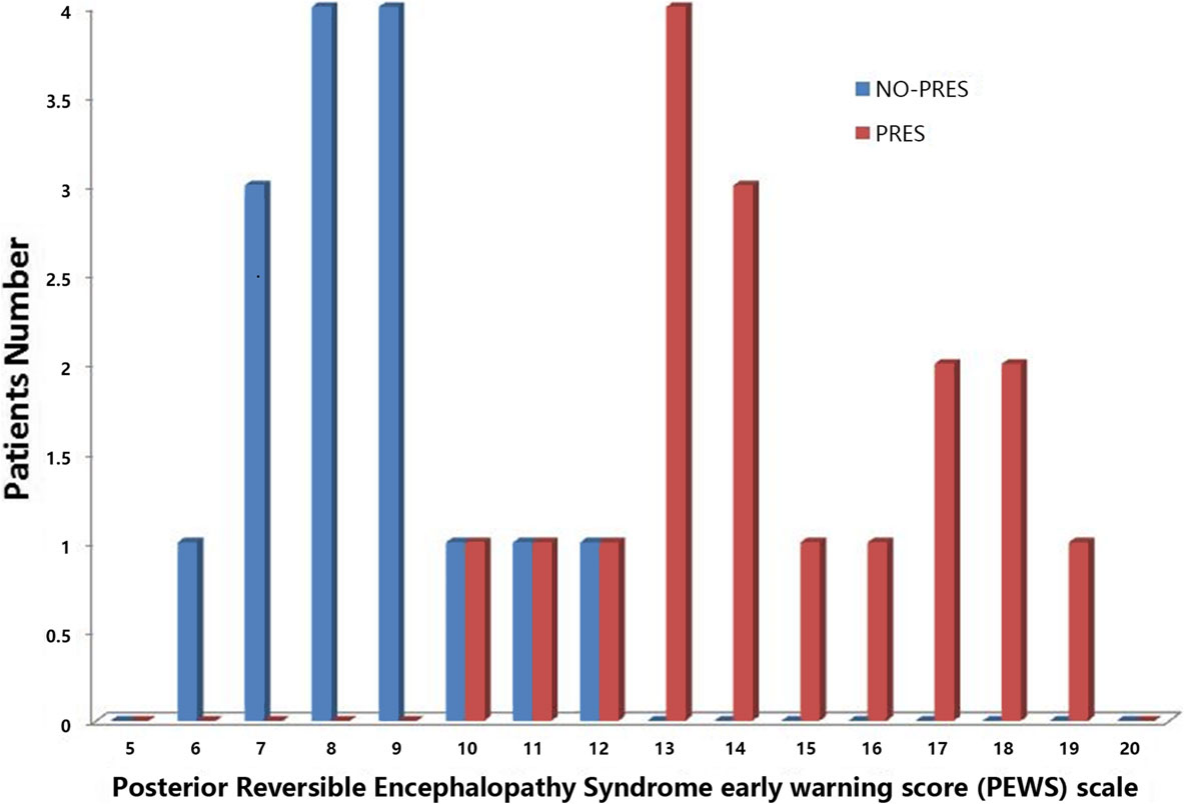
PRES: Posterior reversible encephalopathy syndrome. No-PRES: Encephalitis. Two groups were compared. The median score for the PRES group was 15.5 (range 10–19), and the median score for the No-PRES (Encephalitis) group was 8 (range 6–12)

The PEWS scale demonstrated excellent consistency compared with the gold standard. The median score for the PRES group was 15.5 (range 10–19). The median score for the other encephalopathy group was 8 (range 6–12). The scores of the two groups showed in Fig. 1.

**Fig. 2:**
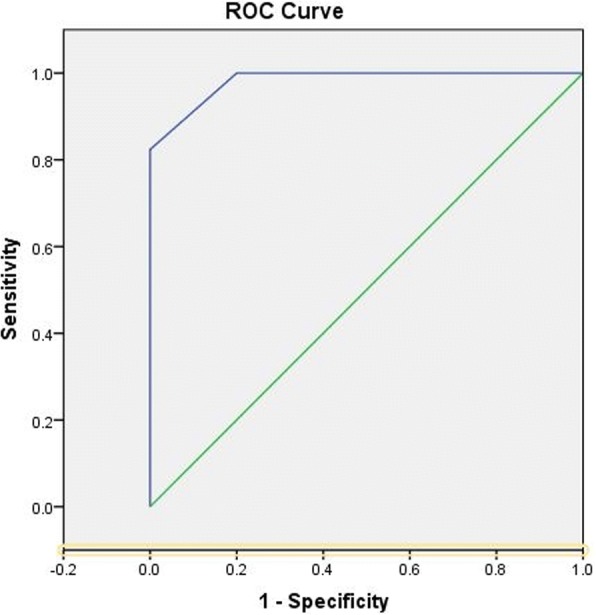
Receiver operating characteristic (ROC) curve of PEWS scale for patients. PEWS scale was able to detect PRES with a sensitivity of 94.1% and a specificity of 86.7%. Area under the curve (AUC) = 0.982, *P* = 0.000, 95%CI: 0.949–1.000

Receiver operating characteristic (ROC) curve of PEWS scale for patients showed in Fig. 2. The Youden Index is 0.824. PRES is a serious disease. Thus, we set the cutoff value to 10 for higher sensitivity. The sensitivity and specificity were 0.941 and 0.867, respectively.

The PEWS scale scores of the two groups presented a partial intersection within a certain range. Thus, we concluded that if the final score is 0–9 points, children are less likely to diagnose with PRES; if the score is 10–12 points, children may diagnosed with PRES; and children with 13 points or above are more likely to diagnose with PRES.

### Part two: Prospective study and the use of PEWS scale

Fifty seven patients with acute lymphoblastic leukemia treated with the VDLP/VDLD program enrolled in this study, including 39 males and 18 females with a median age of 8.7 years old (1–16 years old).

EEG monitoring performed at the beginning of admission, at 2–3 weeks after chemotherapy, and at the end of chemotherapy. The EEG monitoring time of each patient was no less than 2 h. The record should include the awake period, as well as the complete sleep period.

The EEG background activity was normal or nearly normal during initial checking in all patients. EEG examination of the 16 patients during the first 2–3 weeks of chemotherapy suggested a background of slow-wave activity, mainly involving the parieto-occipital lobes (12/16, 75%). Twelve patients exhibited focal δ activity, 3 showed diffused δ and θ activities, and 1 presented focal θ activity (Table 4). PEWS scale scores of these 16 patients shown in Table 5.

**Table 4:** Prospective intervention phase EEG features of 16 patients

	Widespread involvement	Frontal or temporal lobe involvement	Parietal or occipital lobe involvement
delta activity		3 (18.8%)	9 (56.3%)
delta and theta activity	1 (6.3%)		2 (12.5%)
theta activity			1 (6.3%)

**Table 5:** Prospective intervention phase PEWS scale scores of 16 patients

Patient number	Sex/Age(y)	Clinical features (points)	Risk factors (points)	EEG features (points)	PEWS scale (points)	Diagnosis For PRES
1	M/5	3	6	4	13	Yes
2	M/5	3	7	2	12	Yes
3	F/5	0	6	4	10	NO
4	F/7	1	5	2	8	NO
5	M/3	0	6	2	8	NO
6	F/7	1	5	2	8	NO
7	M/7	0	5	3	8	NO
8	F/9	0	5	2	7	NO
9	M/8	0	5	2	7	NO
10	M/9	0	5	2	7	NO
11	M/7	0	5	2	7	NO
12	M/4	0	5	2	7	NO
13	M/10	0	5	2	7	NO
14	M/3	0	5	2	7	NO
15	F/1	0	5	2	7	NO
16	F/5	0	5	1	6	NO

**Fig. 3:**
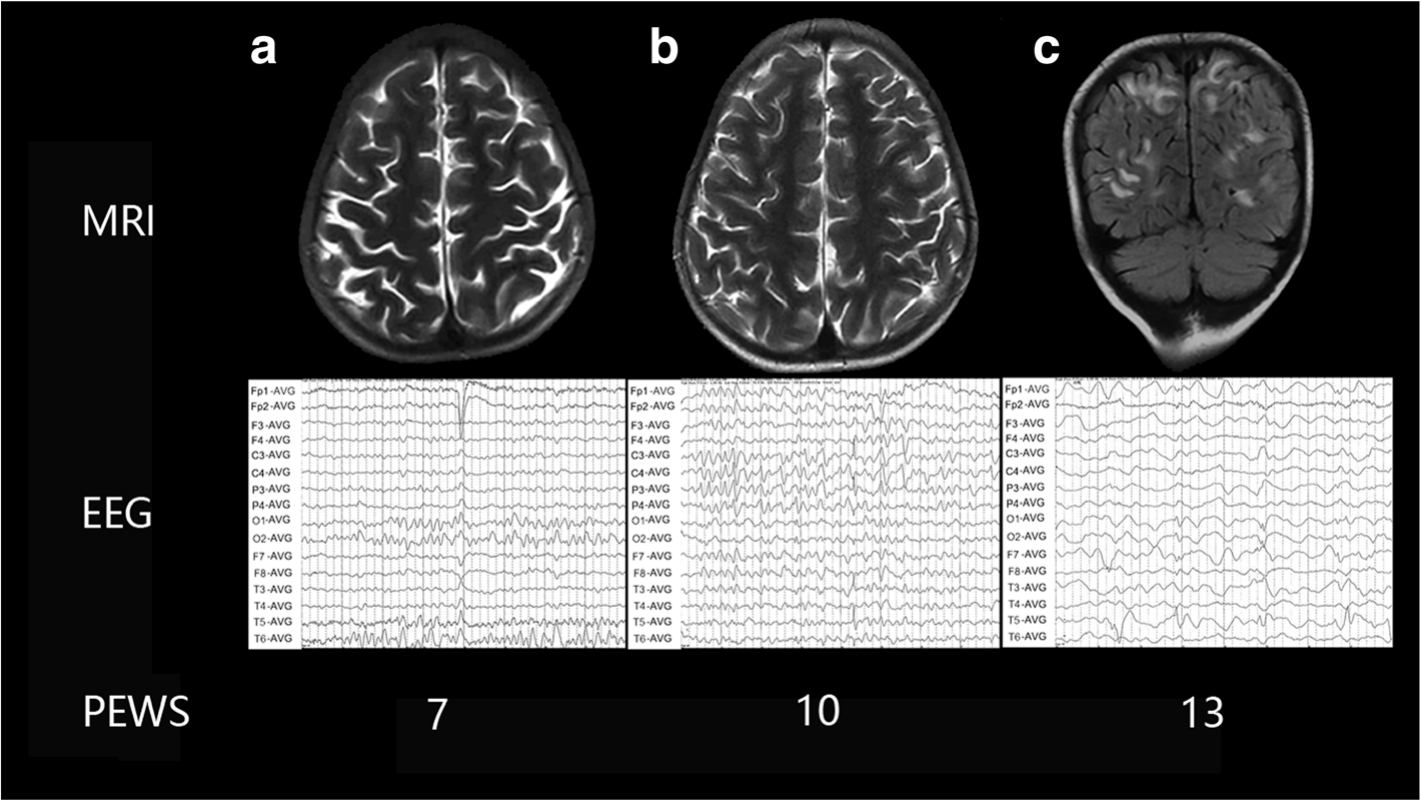
**a** female, five years old, EEG showed focal low amplitude slow wave, MRI was normal, PEWS 7. **b** female, five years old, EEG showed focal (right parietal and temporal lobe) epileptic discharge, MRI was normal, PEWS 10. **c** male, five years old, EEG showed slow wave background and epileptic discharge, MRI showed bilateral parieto-occipital subcortical white matter long T1 signal, long T2 signal, and equal DWI signal, PEWS 13. Abbreviations: EEG: electroencephalogram; MRI: magnetic resonance imaging; PEWS: posterior reversible encephalopathy syndrome early warning score

54 of the 57 patients achieved a PEWS scale score of less than 10 points, and none of the 54 patients diagnosed with PRES. Of the three other patients, one patient with a PEWS score of 10 points presented no significant imaging abnormalities. One patient attained a PEWS scale score of 12, and existed a radiographic presentation of PRES for this patient. After adjusting chemotherapy regimens, the patient did not experience headache, hypertension, seizure, or disturbance of consciousness. One other patient achieved a PEWS scale score of 13 points, suggesting a great possibility of PRES diagnosis. Head MRI findings suggested bilateral parieto-occipital subcortical white matter long T1 signal, long T2 signal, and equal DWI signal, which confirmed to occur PRES. The following day, this patient experienced a single short grand mal seizure. He gave prophylactic intervention, such as antihypertensive treatment, adjustment of chemotherapy, and other intensive management treatments that used before the onset of seizure. The patient achieved complete clinical recovery with subsequent radiologic and EEG resolution in less than 6 months (Fig. 3).

## Discussion

PRES, as a complication during treatment of various diseases, is difficult to diagnose because of its complicated clinical and imaging manifestations. Therefore, clinicians should focus on some atypical radiographic findings. Given PRES significantly varied, and the risk factors and pathogenesis vary. Also, the prognosis of PRES may also differ.

This study performed EEG analysis of patients with PRES. The real significance of the slow-wave performance is the EEG sign of PRES. Although this sign is not specific for PRES, it is an important clue for the diagnosis of this disease.

Oliver et al. [10] reported the EEG features of 17 adult PRES patients in 2011, and abnormal background activities observed in all of them. Diffused θ slowing found in 76.5% of the patients, and 23.5% showed δ activity. We can conclude that, the slow-wave activity in the background is a common occurrence in PRES patients. This activity is an important clue for the diagnosis of this disease. No clear correlation between MRI results and EEG findings has reported among PRES patients.

In our study, 11 patients presented a diffused slow-wave activity in an EEG background; 5 cases (45.5%) confined to the occipital and parietal lobes, as detected by MRI. Other cases (54.5%) affected in the frontal and temporal lobes. This result was consistent with the findings of previous adult studies. EEG is a sensitive and rapid diagnostic technique. EEG results with slow-wave background performance can use for early diagnosis of PRES.

We have demonstrated the PEWS scale was the first research that assessed the reliability of clinical presentation, risk factors, and EEG features for the early diagnosis of PRES.

In the first part of this study, we summarize the risk factors and the clinical, imaging, and EEG features of PRES children. We developed a PEWS scale to help diagnose PRES. In the second part of our study, the scale validated to clarify the effect of the PEWS scale. Considering those different diseases that induce PRES present different prognoses, we specifically selected acute lymphoblastic leukemia patients receiving induction chemotherapy as subjects in the second part of our study. The patients in this period presented a variety of risk factors that induced PRES. The clinical and imaging features of concurrent PRES were often atypical and demonstrated poor prognosis. To address all the elements of this study and to avoid interfering different underlying diseases, we used these patients as our research subjects to be as representative as possible. In the second part, 57 children with leukemia evaluated using the PEWS scale. According to assessing of the patients’ condition and adjustment of treatments, only one patient with PRES presented mild symptoms and rapid recovery without leaving any sequelae.

**Fig. 4:**
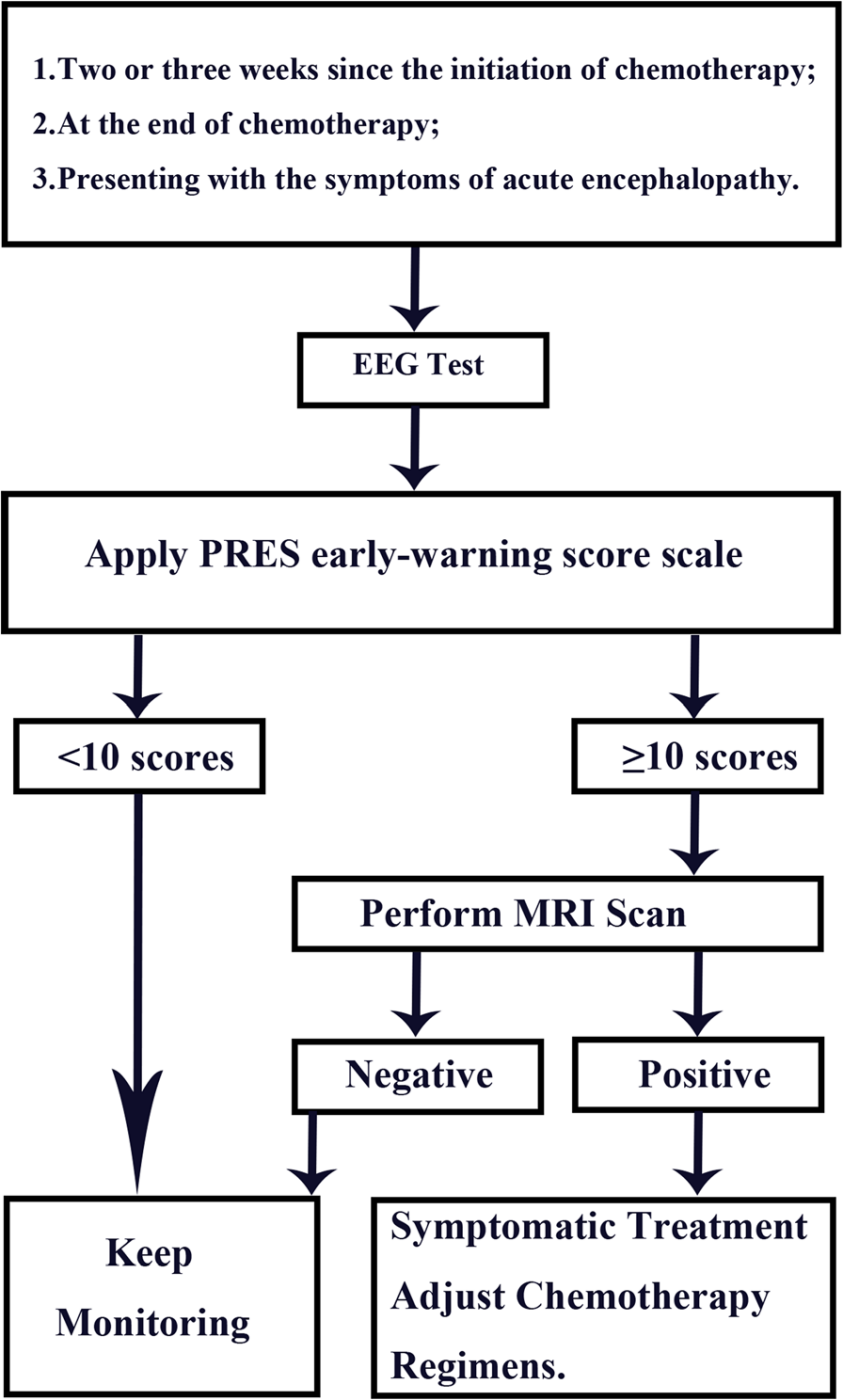
The flowchart of PRES early warning score scale

MRI is the gold standard for the diagnosis of PRES, and practitioners need to decide whether MRI scan should give. PEWS scale is an effective and operable tool for early warning of PRES and assessment of its severity. This scale has a wide range of applications. Clinicians should consider PRES when PEWS scale scores are more than 10 points, and they should perform MRI scan for diagnosis. This will help with selecting different levels of prophylactic treatments to reduce the incidence and to improve the prognosis of patients with PRES. We designed the flowchart of PRES early warning scoring scale to help clarify its use (Fig. 4).

The low prevalence of PRES was the reason that we only found two patients with this condition. Now, in our center, PEWS has been used as a routine assessment of patients with acute encephalopathy and hematologic malignancies. After publishing of the PEWS scale, we hope that more pediatricians can apply it to diagnose and provide intensive intervention in patients with suspected PRES.

PEWS scale successfully applied to our 57 patients, a larger sample size and a multi-center collaborative study. But our study required for further clinical validation.

## Conclusions

Seizures are the most common clinical features among children with PRES. Children with hematological malignancies present poor prognosis. Slow-wave background activity is a common EEG manifestation of PRES, and its appearance is a significant clue for occurs of this disease. PRES highly suspected when the PEWS scale score is 10 or higher, and pediatricians need to focus more on this condition.

## Abbreviations

EEG: Electroencephalogram
HSCT: Hematopoietic stem cell transplantation
MRI: Magnetic resonance imaging
PEWS: Posterior reversible encephalopathy syndrome early warning scoring
PRES: Posterior reversible encephalopathy syndrome
